# Supplementary material for: The causal role of circulating inflammatory markers in osteoporosis: a bidirectional Mendelian randomized study
Source: Front Immunol. 2024 Jul 18;15:1412298. doi: 10.3389/fimmu.2024.1412298 (PMC11291241; doi:10.3389/fimmu.2024.1412298)
Supplement: Supplementary file 1 [file DataSheet_1.zip › supplementary material/OSTEOPOROSIS-CIM/GCST90274764.tsv.gz_leave_one_out.pdf]

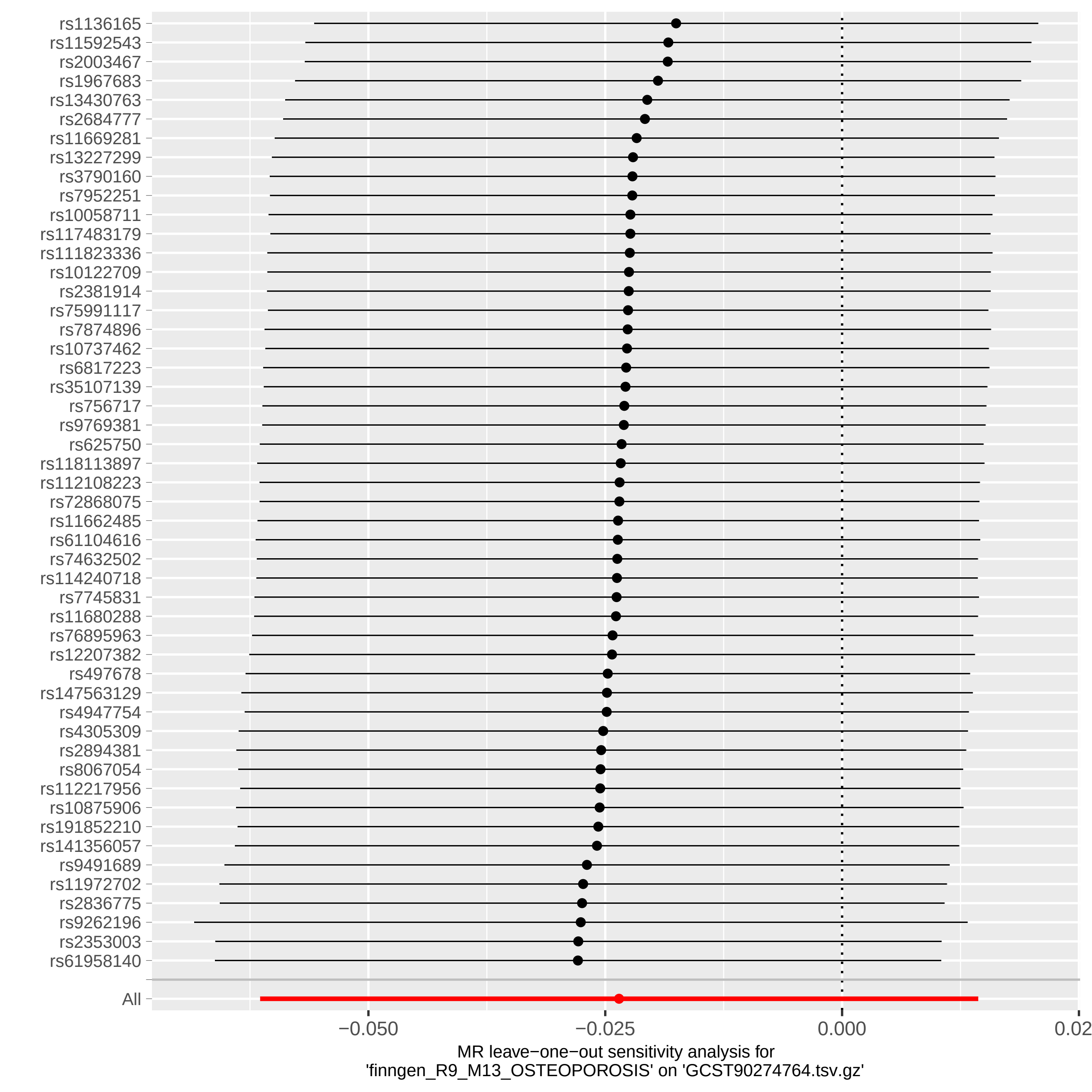

rs1136165
rs11592543
rs2003467
rs1967683
rs13430763
rs2684777
rs11669281
rs13227299
rs3790160
rs7952251
rs10058711
rs117483179
rs111823336
rs10122709
rs2381914
rs75991117
rs7874896
rs10737462
rs6817223
rs35107139
rs756717
rs9769381
rs625750
rs118113897
rs112108223
rs72868075
rs11662485
rs61104616
rs74632502
rs114240718
rs7745831
rs11680288
rs76895963
rs12207382
rs497678
rs147563129
rs4947754
rs4305309
rs2894381
rs8067054
rs112217956
rs10875906
rs191852210
rs141356057
rs9491689
rs11972702
rs2836775
rs9262196
rs2353003
rs61958140
All
−0.050
−0.025
0.000
0.02
MR leave−one−out sensitivity analysis for
'finngen_R9_M13_OSTEOPOROSIS' on 'GCST90274764.tsv.gz'
